# Mental Health Screening Needs and Preference in Treatment Types and Providers in African American and Asian American Older Adults

**DOI:** 10.3390/brainsci11050597

**Published:** 2021-05-05

**Authors:** Minsun Lee, Wenyue Lu, Tyrell Mann-Barnes, Jin-Hyeok Nam, Julie Nelson, Grace X. Ma

**Affiliations:** 1 Center for Asian Health, Lewis Katz School of Medicine, Temple University, 3440 N. Broad Street, Philadelphia, PA 19140, USA; minsun.lee@temple.edu (M.L.); wenyue.lu@temple.edu (W.L.); tyrell@temple.edu (T.M.-B.); jnam@temple.edu (J.-H.N.); 2 Philadelphia Senior Center, Philadelphia, PA 19147, USA; jnelson@philaseniorcenter.org; 3 Department of Clinical Sciences, Lewis Katz School of Medicine, Temple University, 3500 N. Broad Street, Philadelphia, PA 19140, USA

**Keywords:** mental health providers, treatment preference, African Americans, Asian Americans, older adults

## Abstract

Older African Americans and Asian Americans in the U.S. underuse mental health services, despite their vulnerability to diverse mental health problems. This study examined their perspectives on the importance of various mental health problems, mental health treatment, and provider type preference. A total of 243 participants residing in Philadelphia were recruited through community-based organizations. Chi-square, ANOVA, and logistic regression were conducted to examine ethnic differences in demographic characteristics, mental health screening needs, and treatment preferences. African Americans were more likely to endorse the screening needs for depression (AOR: 3.77; 95% CI: 1.19–11.93, *p* < 0.05) and less likely to endorse the screening needs for suicide (AOR: 0.24; 95% CI: 0.08–0.76, *p* < 0.05) compared to Asian Americans. For treatment preferences, African Americans were more likely to seek help from primary care physicians (AOR: 8.26; 95% CI: 1.71–32.86, *p* < 0.01) and less likely to prefer medication as a treatment option (AOR: 0.36; 95% CI: 0.09–0.79, *p* < 0.05) than Asian Americans. African Americans and Asian Americans prioritized mental health screening needs differently and had different treatment preferences, indicating that matching community needs and preferences regarding mental health services is critical to improve mental service utilization rates in the targeted populations.

## 1. Introduction

Compared to non-Hispanic white communities, communities of ethnic minorities significantly underutilize mental health services [1,2]. In particular, Asian Americans have the lowest rates of mental health service utilization in the United States [3,4] after controlling for age, gender, and education. Asian Americans are also likely to delay help-seeking and utilizing inpatient services when symptoms become more severe [2,5,6]. Individuals who do seek help often prefer general health providers and informal services instead of mental health professionals [7,8,9,10] and have higher attrition rates after initiating mental health service treatment [2,3,6,7,9].

Based on data from the National Survey of American Life, one study found that the 12-month mental health utilization rate among African Americans 18 years and older was as low as 10.1% [11]. Data from a survey of Baltimore residents aged 18 or older indicated that 22% of African Americans sought informal mental health care from spiritual leaders, social service organizations, and family members; this rate was similar to the rate of people who utilized formal mental health services [12]. In addition, African Americans are reported to have a more negative attitude toward seeking mental health treatment, compared to whites [13]. Low levels of help-seeking behavior have been associated with untreated illness, lower quality of life [14], higher mortality rate [15], shorter life expectancy [16], and increased vulnerability for future onset of mental illness. 

The underutilization of mental health services among Asian Americans and African Americans does not stem from a low prevalence of mental health problems or a lack of need for services. Compared to the general population, African Americans have higher rates of depression [17], substance use disorders [18], and post-traumatic stress disorder (PTSD) [19]. Although Asian Americans are reported to have a lower prevalence of mental health problems than the general population [19], they are more likely to develop specific mental health problems, including but not limited to suicidal ideation [20]. Compared with whites, older Asian and African Americans in California reported higher rates of psychological distress, with increased levels of severity [21]. Moreover, even when levels of mental health problems are similar to levels among whites, both African and Asian Americans are less likely to seek professional help [22]. 

Racial gaps in the use of mental health services, despite the demonstrated need for prevention and treatment, have been attributed to structural, cultural, and systemic barriers experienced by ethnic minorities [7,23]. For example, lack of financial resources and health insurance, limited English proficiency, and inadequacy of culturally appropriate mental health services significantly contribute to reduced access to needed treatment in ethnic minority groups [7,24]. Stigma and negative attitudes toward mental health services are other common barriers that prevent service utilization across ethnic minority groups [25,26]. Moreover, acculturation and immigration-related factors significantly influence Asian Americans’ mental health service utilization [3,16,27,28], indicating a need for culturally adapted treatment [29].

The older population in the United States is another group that is vulnerable to having untreated mental health issues. The population of Americans aged 65 or older has increased by 33%; the size of this population is predicted to double to 98 million by 2060 [26]. Epidemiologists predict that the number of older adults living with mental disorders, such as mood, anxiety, or psychiatric disorders, will increase to approximately one in five older adults by 2030 [25,30]. Moreover, the elderly are more likely than young adults to stigmatize mental illness and therefore are less likely to seek help [31]. In addition to pervasive stigma, other prominent barriers to seeking help for mental health problems among elderly individuals include low awareness of mental health problems, lack of social support, and shortage of certified geriatric mental health specialists [14,25]. Older minority adults with mental illnesses experience a “triple stigma”—being advanced in age, being in a minority group, and having reduced access to services—which leaves them especially vulnerable [31]. Considering the projected increase in older minority populations, in conjunction with observed low rates of mental health service use in this group, it is critical to explore the needs and preferences of older minorities to ensure their mental health in the future.

Although research has increased knowledge on barriers to and facilitators of mental health-seeking behaviors among diverse groups, few studies have examined the influence of treatment and provider preference on these behaviors. There is increasing evidence that it is important to consider treatment preference when deciding on the treatment approach for individuals with mental health problems. For example, studies have demonstrated that Americans with mental disorders have treatment preferences for psychotherapy over pharmacological treatment or psychotherapy in combination with medication over medication alone [32,33,34]. The difference in treatment preferences is linked to the patient’s personal belief on the etiology of his or her disorder. Individuals who prefer psychotherapy may believe that their depression is from psychosocial stress, while those who prefer medications may believe their depression is from a chemical imbalance [35]. Studies have shown that matching depression patients to preferred treatment modalities (psychotherapy vs. medication) is associated with decreased dropout from therapy [36]. Minorities tend to prefer mental health providers who racially or ethnically match their own race or ethnicity [1]. Among Asian American children and adults, patient-provider matching appears to be effective in improving mental service utilization, customer satisfaction, and treatment outcomes [1,37,38,39].

Evidence suggests that matching treatment and provider preference can potentially increase mental health service initiation and treatment adherence rates. Further, understanding mental health treatment and provider preferences among older African and Asian American adults can improve mental health care utilization [40,41] and treatment outcomes [42]. Therefore, the purpose of this study was to examine: (1) which mental health problems are perceived as important issues that require prevention and early detection in African and Asian American older adults, and (2) whether African and Asian Americans older adults have similar or different preferences in mental health treatment and provider types.

## 2. Methods

### 2.1. Participants

A total of 243 older adults (aged 50 years or older) residing in the greater Philadelphia area were enrolled in the study. Among participants, 93 were African Americans, 43 were Chinese Americans, 42 were Korean Americans, and 65 were Vietnamese American. Based on a long-term collaboration history, community-based organizations (CBOs), including churches, senior centers, and cultural organizations, in the greater Philadelphia area were contacted for the recruitment of eligible participants.

### 2.2. Data Collection

Collaboration between the Center for Asian Health at Temple University and participating CBOs was initiated to facilitate recruitment and data collection. CBO leaders contributed to the recruitment process, working together with the research team to identify eligible participants, providing detailed information about the study, obtaining informed consent, and administering surveys.

### 2.3. Measures

All questionnaires were administered in English, Korean, Mandarin, and Vietnamese languages for participants who preferred to use their heritage languages. Demographic and background information included age, ethnicity, gender, marital status, educational level, and family income. Through the survey questions, participants reported their demographic information: age (date of birth), ethnicity (African American or Chinese, Korean, or Vietnamese American), sex (male or female), marital status (single, married, or divorced/separated/widowed), education (completed high school or under, college or above), and family income (less than USD 40,000, USD 40,000–75,000, or more than USD 75,000 per year). Marital status was coded as “currently single” or “married” for the main analysis. In addition to demographic information, two questions were asked to assess health-related behaviors: one question asked whether the participants saw their primary care physician (PCP) for a routine checkup or any health issue in the last 12 months, and the other question asked whether the participant’s PCP asked about any mental health problems, including depression, anxiety, or drug and alcohol use during the visit.

The need for screening was assessed by asking participants whether they believed it would be helpful to provide a screening test for different types of mental health problems in their community. The listed psychological disorders included depressive disorder, PTSD, panic disorder, alcohol or drug abuse, and suicidal risk. Each disorder was rated on a Likert-type response scale of 1–4, ranging from “not helpful at all (1)” to “very helpful (4)”. The average score for each disorder was used for analysis.

Preference on the type of treatment received was assessed by listing different types of psychological treatments (medication, psychotherapy, a combination of medication and psychotherapy, telephone or internet-based psychotherapy, self-help, and others). Respondents marked types of treatments that they would prefer to receive.

Preference for mental health providers was assessed by asking participants who they would like to go to if they wanted to receive help for their mental health problems. Options included a primary care physician (PCP), psychiatrist, psychologist/counselor, spiritual leader (e.g., pastor), friends, or relatives.

### 2.4. Data Analysis

Descriptive statistics were used to examine the frequency and mean (standard variation) of variables of interest. Chi-square analysis and ANOVA were conducted to examine ethnic differences in categorical variables (e.g., demographics, preferred treatment types, and providers) and continuous variables (e.g., mean scores of screening needs for each disorder), respectively. To address the main research questions, the differences in screening needs and treatment preferences between African and Asian Americans, logistic regression analyses were conducted with ethnicity being a dependent variable.

## 3. Results

### 3.1. Demographic Characteristics of Participants

As shown in Table 1, the mean age for the participants was 67.6 years old. Women (63.7%) represented the majority of the participants. In the African American group, this difference was more pronounced, with women representing 71.6% of the sample and men representing 28.4%. In terms of marital status, 52.5% of the total sample reported being married. However, whereas more than three-fourths of Asian Americans were married (75.3%), the majority of African American participants reported being single (84.8%), indicating significant ethnic differences in marital status (χ^2^ (1, *n* = 242) = 87.64, *p* < 0.001). Overall, 57.2% of participants had a high school education or less, with similar rates in both African and Asian American groups. Family income did not show a significant difference between the two ethnic groups, with 81.8% of the total participants reporting an income of less than USD 40,000 per household.

### 3.2. Perceived Needs for Screening Mental Health Problems in African and Asian Americans

Table 1 shows scores of screening needs for prevention and early detection of mental health problems in African and Asian Americans. For different mental health problems, African Americans reported that screening for drug and alcohol use would be most helpful (3.15), followed by major depressive disorder (3.14), PTSD (3.11), panic disorder (3.07), and suicidal thoughts (3.03). Asian Americans reported that screening for suicidal thoughts would be most helpful (3.13), followed by drugs and alcohol use (3.10), panic disorder (2.99), major depression disorder (2.90), and PTSD (2.85). Results of logistic regression analysis (Table 2) revealed that after controlling for age, gender, marital status, education, and family income, African Americans were more likely to endorse the screening needs for depression (AOR: 3.77; 95% CI: 1.19–11.93, *p* < 0.05) and less likely to endorse the screening needs for suicide (AOR: 0.24; 95% CI: 0.08–0.76, *p* < 0.05) compared to Asian Americans.

### 3.3. Ethnic Differences in Preference of Treatment Providers and Types

As presented in Table 2, after controlling for demographic variables, African Americans were significantly more likely to seek help from PCPs (AOR: 8.26; 95% CI: 1.71–32.86, *p* < 0.01) compared to Asian Americans. Although not statistically significant (*p* < 0.05), African Americans also tended to seek help more from psychologists and psychiatrists (AOR: 2.99; 95% CI: 0.91–17.57, *p* = 0.06) than Asian Americans. With regard to the preference of treatment type, compared to Asian Americans, African Americans were significantly less likely to prefer medication (AOR: 0.36; 95% CI: 0.09–0.79), *p* < 0.05) as a treatment option.

## 4. Discussion

This study examined perceived mental health screening needs and preferences in treatment and provider types in African and Asian American older adults. Among older Asian Americans, suicide was listed as the most prioritized mental problem, followed by drug and alcohol use problems. Among older African Americans, however, drug and alcohol use problems were reported to be the most important issues, followed by depression. Suicide was identified as the least important problem for screening by older African Americans. These results are consistent with previous studies about the main mental health problems in African and Asian American communities. In 2016, suicide was the 10th leading cause of death for Asian Americans and the 16th leading cause of death for all racial groups in the United States [20]. U.S.-born Asian American women have reported a higher lifetime rate of suicidal thoughts (15.9%) than that of the general U.S. population (13.5%) [43]. Moreover, studies have shown that elderly Asian Americans have some of the highest rates of suicidal thoughts and behaviors, while older African Americans have the lowest rate (56.8% vs. 27%) [44]. Asian Americans in our study appear to be well aware of the problems with suicide in their community and endorse the need for suicide screening.

Addiction is a highly prevalent problem among older adults in general [45] and was recognized as an important issue by both Asian and African Americans in this study. Asian Americans generally have low rates of substance use disorder [46]. However, they have a high frequency of alcohol consumption and experience multiple barriers to substance use treatment, which explains the low rates of treatment for substance use previously observed among Asian Americans [47]. Although older African Americans have lower alcohol consumption than whites [48], they have the highest rate of illicit drug use [18]. African Americans reported depression as the second most important mental illness, while Asian Americans were less concerned about the prevalence of depression in their community. In fact, depression has been reported to be more common among African Americans, with a 9.2% prevalence rate, compared to a 3.1% prevalence rate among Asian Americans [17]. Our findings, consistent with the aforementioned prevalence data, indicate that older African American adults have a greater awareness of the prevalence of depression in their community in comparison to Asian Americans.

For both African and Asian Americans, PCPs are an important source to prevent, recognize, and treat mental illness [49]. PCPs frequently serve as a bridge between patients with mental disorders and mental health specialists, including psychiatrists and psychologists [50]. In this study, we found that African Americans significantly preferred to receive mental health treatment from PCPs compared to Asian Americans. This finding is consistent with previous studies [51,52,53], in which older African Americans with indications for mental illnesses, compared to Asian Americans with similar indications, reported feeling more comfortable communicating their problems to medical professionals [26]. This finding is also supported by our study, which demonstrated that, compared to PCPs who see Asian Americans, PCPs serving African Americans are significantly more likely to discuss mental health problems with their patients. This result indicates that both African American older adults and their PCPs are more open to discussing mental health-related topics than Asian Americans and their PCPs. Thus, the lower preference of PCP as a mental health treatment provider among Asian American older adults may be partly due to the limited availability of PCPs who are willing to discuss mental health issues with their Asian patients [54]. Other potential explanations may involve unique expectations of Asian Americans. For example, Asian Americans tend to prefer older mental care providers who share similar beliefs and values with them and who are highly knowledgeable in the field [54,55]. These expectations may prevent older Asian American adults from discussing their mental health problems with PCPs who they consider an expert of physical illness but less competent in Asian cultural values.

Another important finding of this study is that older Asian Americans reported a more favorable attitude toward medication as a mental health treatment method than African Americans. Asian and African Americans, however, were not significantly different in their preference of psychotherapy and combined medication and psychotherapy. Previous studies have demonstrated that both Asian and African Americans with mental disorders, including depression and panic disorders, were less likely to endorse pharmacotherapy than their white age-matched counterparts [56,57,58]. However, in our study, older African Americans, compared with older Asian Americans, were more likely to accept medications as the sole treatment option. Potential explanations for these differences include limited English proficiency and greater stigmatization toward receiving psychiatric or psychological treatment among Asian American older adults.

Limited English proficiency has been significantly associated with low mental health service utilization [59]. This may be partly because verbal communication is essential in the diagnosis and treatment of mental health problems. With limitations in both English proficiency [59] and access to bilingual mental health professionals [54], older Asian Americans would prefer medication as a treatment option. As a treatment modality, medication requires fewer verbal skills and that reduced cultural stigma. For many Asian Americans who consider receiving psychiatric treatment to be shameful [1,54,60,61], using prescribed medications can reduce the frequency of psychotherapist visits and lower the risk of disclosing their mental health status to others.

This study had several limitations. We included only three Asian subgroups in the United States: Chinese, Korean, and Vietnamese Americans. Although major depressive disorder and PTSD were not major concerns for our Asian sample, they might have been identified as major problems for other groups, such as Cambodian refugees, in whom the prevalence rates of major depression (62%) and PTSD (51%) are high [62]. Therefore, caution should be exercised in generalizing the findings of this study to other Asian subethnic groups. Because many previous studies have compared mental health service utilization patterns between non-Hispanic whites and aggregated minority populations, efforts must be made to better understand the unique needs and treatment preferences in each ethnic minority group. Another limitation is that this study did not assess whether participants prefer providers who have the same ethnicity or gender. It was reported previously that a racial/ethnic match generated better treatment outcomes among ethnic minorities [63]. Thus, future studies about the impact of patient-physician ethnicity and/or gender concordance on mental health treatment outcomes are necessary and will be informative. Continuing efforts to identify unique mental health issues and the factors that can enhance mental health service use in different subgroups of Asian Americans and Black/African Americans, as well as other ethnic groups, are warranted. Finally, studies about the implementation of targeted mental health screening and follow-up management are necessary.

## 5. Conclusions

The findings of this study underline the importance of understanding distinctive needs and preferences for mental health treatment options in different ethnic and cultural groups. Specifically, our findings imply that PCPs should be more aware of the mental health problems facing their patients and make appropriate efforts to discuss mental health issues with patients, particularly African and Asian American older adults, during routine care. Moreover, community education and health promotion are necessary to increase knowledge about different mental health treatment options and enhance communication with PCPs about emotional and mental health concerns in both populations. Policymakers also need to focus on increasing the availability of culturally and linguistically appropriate mental health professionals and on promoting cross-cultural community education initiatives to increase engagement of medical interpreters in mental health consultation for those who have limited English abilities.

## Figures and Tables

**Table 1:** Participant characteristics by ethnic groups.

Variables	Total (*n* = 243)	African Americans (*n* = 93)	Asian Americans (*n* = 150)	χ^2^, F
	M (SD), %	M (SD), %	M (SD), %	
Age	67.6 (9.40)	68.4 (9.67)	67.11 (9.23)	1.03
Gender				3.76
Male	86 (36.3%)	25 (28.4%)	61 (40.7%)	
Female	151 (63.7%)	63 (71.6%)	88 (58.7%)	
Marital Status				82.64 ***
Currently Single	115 (47.5%)	78 (84.8%)	37 (24.7%)	
Married	127 (52.5%)	14 (15.2%)	113 (75.3%)	
Education				4.38 *
High school or under	135 (63.1%)	51 (71.4%)	84 (57.9%)	
College or more	101 (36.9%)	40 (28.6%)	61 (42.1%)	
Family Income				0.61
Less than USD 40,000	180 (81.8%)	66 (82.5%)	114 (81.4%)	
USD 40,000 or more	40 (18.2%)	14 (17.5%)	26 (18.6%)	
Health check with PCP in last 12 months	212 (89.8%)	86 (92.5%)	126 (88.1%)	1.17
Discussion on mental health with PCP	78 (34.7%)	53 (60.9%)	25 (18.1%)	43.17 ***
**Need for Screening**				
MDD	3.00 (0.84)	3.14 (1.04)	2.90 (0.66)	4.51 *
PTSD	2.95 (0.88)	3.11 (1.10)	2.85 (0.69)	4.49 *
Panic Disorder	3.02 (0.85)	3.07 (1.06)	2.99 (0.69)	0.43
Drug and Alcohol Use	3.11 (0.92)	3.15 (1.15)	3.10 (0.76)	0.16
Suicidal thoughts	3.09 (0.91)	3.03 (1.20)	3.13 (0.71)	0.56
**Treatment Type**				
Medication	57 (25.1%)	20 (23.0%)	37 (26.4%)	0.34
Psychotherapy	67 (29.5%)	32 (36.8%)	35 (25.0%)	3.58
Medication and psychotherapy	57 (25.1%)	19 (21.8%)	38 (27.1%)	0.80
Treatment Providers				
PCP	92 (39.7%)	48 (54.5%)	44 (30.6%)	13.14 ***
Psychiatrist/psychologist	90 (38.8%)	35 (39.8%)	55 (38.2%)	0.057
Spiritual leaders/friends	70 (30.2%)	25 (28.4%)	45 (31.3%)	0.209

Note: * *p* < 0.05; ** *p* < 0.01; *** *p* < 0.001.

**Table 2:** Ethnic differences in screening needs and mental health treatment and provider types.

Variables	African vs. Asian Americans	
	AOR (95% CI)	AOR (95% CI)
Age	1.00 (0.96–1.05)	1.01 (0.95–1.04)
Gender	0.97 (0.39–2.43)	1.52 (0.68–3.96)
Marital Status	0.03 (0.01–0.09) ***	0.05 (0.01–0.11) ***
Education	0.71 (0.27–1.85)	0.92 (0.59–3.68)
Family Income	2.75 (0.61–7.11)	1.92 (0.77–3.62)
Screening Needs		
Depression	3.77 (1.19–11.93) *	
Panic disorder	0.80 (0.24–2.70)	
PTSD	0.94 (0.34–2.61)	
Drug and Alcohol Use	2.33 (0.87–6.22)	
Suicide	0.24 (0.08–0.76) *	
**Provider Type**		
PCP		8.26 (1.71–32.86) **
Psych. and Psych.		2.99 (0.91–17.57)
Spiritual leader and Friend		2.48 (0.83–10.88)
**Treatment Type**		
Medication		0.36 (0.09–0.79) *
Psychotherapy		1.27 (0.41–2.80)
Combination		0.57 (0.17–1.61)

Note: (1) AOR: adjusted odds ratio, CI: confidence interval. (2) * *p* < 0.05; ** *p* < 0.01; *** *p* < 0.001.

## Data Availability

The data are not publicly available due to confidentiality.
